# Combined Manufacturing Process of Copper Electrodes for Micro Texturing Applications (AMSME)

**DOI:** 10.3390/ma14102497

**Published:** 2021-05-12

**Authors:** Carlos J. Sánchez, Pedro M. Hernández, María D. Martínez, María D. Marrero, Jorge Salguero

**Affiliations:** 1 Departamento de Ingeniería Mecánica, Campus de Tafira, Universidad de Las Palmas de Gran Canaria, Parque Científico Tecnológico ULPGC, 35017 Las Palmas de Gran Canaria, Spain; pedro.hernandez@ulpgc.es (P.M.H.); mariadolores.martinez@ulpgc.es (M.D.M.); mariadolores.marrero@ulpgc.es (M.D.M.); 2 Departamento de Ingeniería Mecánica y Diseño Industrial, Universidad de Cádiz, Avenida de la Universidad de Cádiz, 10, 11510 Puerto Real, Spain; jorge.salguero@uca.es

**Keywords:** structured surfaces, texturing, micro-electroforming, additive manufacturing, sinker electro discharge machining

## Abstract

Surface texturing has brought significant improvements in the functional properties of parts and components. Sinker electro discharge machining (SEDM) is one of the processes which generates great texturing results at different scale. An electrode is needed to reproduce the geometry to be textured. Some geometries are difficult or impossible to achieve on an electrode using conventional and even unconventional machining methods. This work sets out the advances made in the manufacturing of copper electrodes for electro erosion by additive manufacturing, and their subsequent application to the functional texturing of Al-Cu UNS A92024-T3 alloy. A combined procedure of digital light processing (DLP) additive manufacturing, sputtering and micro-electroforming (AMSME), has been used to produce electrodes. Also, a specific laboratory equipment has been developed to reproduce details on a microscopic scale. Shells with outgoing spherical geometries pattern have been manufactured. AMSME process has shown ability to copper electrodes manufacturing. A highly detailed surface on a micrometric scale have been achieved. Copper shells with minimum thickness close to 300 µm have been tested in sinker electro discharge machining (SEDM) and have been shown very good performance in surface finishing operations. The method has shown great potential for use in surfaces texturing.

## 1. Introduction

One of the biggest technological challenge in modern engineering is trying to reduce the negative effects caused by the wear phenomenon. This is a problem which is relevant to the lifetime of metallic parts. Some studies have demonstrated that surface texturing [1] decreases friction and improves the tribological performance of mechanical components. This reduces their operating temperatures and increases component life [2]. Hamilton et al. already proposed in 1966 to reduce the friction of a mechanical seal by applying a texture pattern to the surface [3]. The scientific community have made many efforts to do research and evolve in this field [4].

High and low relief geometries can be used to modify the topography of a material. Engraving shapes usually require top-down processes based on material removal from the surface (cutting, grinding, etching, laser). Emboss shapes use bottom-up processes based on the addition of the material on the surface (anodic oxidation of aluminum, self-assembly of particles, block copolymer, electroforming).

When this modification is made with a defined structure or pattern, structured surfaces are achieved [5]. For example, by modifying surface roughness levels, super hydrophobic surfaces can be obtained [6]. These are very interesting for self-cleaning applications and improving aerodynamic resistance. The improvement of aesthetic, adhesion, heat dissipation, hydrodynamics and optical behavior of surfaces [7] are more examples of applications for functional texturing. However, neither the design of the texture nor the process to obtain it are standardized.

Observation is used to find good functional surface solutions: A lotus leaf (hydrophobic); a lizard’s foot (adhesion); a moth eye (antireflective) are some examples of great texturing designs created by Mother Nature [8]. 

Conventional machining or chemical machining processes were traditionally used for surface texturing. At the beginning, drilling, milling, and turning were the most used ones. Later, numerical control and ultrasound vibration systems increased the accuracy and precision of these processes. The controlled chemical dissolution of the machined workpiece material by contact with a strong chemical reagent [9], was already used in the 1950s to remove material from a surface [10]. Today, processes such as jet electrochemical machining (Jet-ECM) are exponents of flexibility and precision [11]. 

Micro-structured or micro-texturized surfaces are obtained due to these technological advances to generate functional patterns. The micro cavities patterns have been particularly interesting for this work development [12]. In many cases, complex geometries with high detail reproduction do not allow the use of conventional machining processes in materials with different harnesses. To do this, at different dimensional levels, it is necessary to use unconventional machining techniques, such as electro discharge machining (EDM) and laser techniques. These techniques have a specific application to the micro texturing of surfaces such as micro-electrical discharge texturing (EDT) or laser surface texturing (LST). There are several studies of the application of these techniques [13,14,15].

In micro EDM, material removal is due to electrical discharge between the tool (electrode) and the work piece through a dielectric fluid [16]. These high voltage discharges create enough thermal energy to cause workpiece erosion. Work piece and electrode are not in physical contact, so no stress is generated, and tool deformation is avoided. This allows the use of small tools suitable for micro machining [17]. The EDM process completes complex geometries with high dimensional accuracy and low roughness surfaces. It does not affect mechanically the surface of the work piece, and it can be used for both, large and small surfaces. 

Two Russian scientists, Lazarenko and Lazarenko at Moscow University were the first to develop a controlled process for machining materials [18]. From them, a growing interest was generated in the novel applications of the process, with special emphasis on its ability to modify surfaces [19]. In recent decades, its application has been explored, specifically, to reduce wear. Dong et al. used a micro EDM system with a cylindrical micro tool to generates a micropatterned insert. They achieved to decreases cutting force in hard turning machining [20]. Koshy and Tovey used EDM to texturize a finish-ground cutting tool. They compared a linear texture with grooves 100 µm depth and with and an areal texture on tool surface. They stablished that such textures are effective in reducing friction, due to significant reduction in machining force. The result was better for areal one, however they proposed to developed a hybrid texture that integrates linear and areal textures to further improve lubricant’s performance [21]. However, surface functional texturing remains poorly studied for other applications.

They use different geometries such as groove, dimple, or channel. The use of dimples on the surface of golf balls is well known. Its application modifies the aerodynamics of the surface and improves its flight. Examples of used dimple geometries are: circular [22], elliptical [23], square [24], triangle [25], rectilinear or diamond shaped [26]. 

Geometries forming depends on the abilities of the manufacturing process. For example, conventional machining processes cannot create negative semi spheres without altering the surface. Also, they cannot create electrodes with positive semi spheres, except by electro-deposition. The sinker electro discharge machining process (SEDM) can generate some of these geometries on a micrometric scale. 

A combined process with DLP Additive Manufacturing, Sputtering and Micro Electroforming (AMSME) has been tried to manufacture SEDM tools.

DLP and micro-electroforming are two additive manufacturing processes with proven results in detail reproductions. In addition, an intermediate process was added as a link between both technologies. Sputtering transforms the polymeric parts manufactured by DLP into conductive parts of the electric current. Therefore, they can be used as a cathode in the electroforming process.

**Figure 1:**
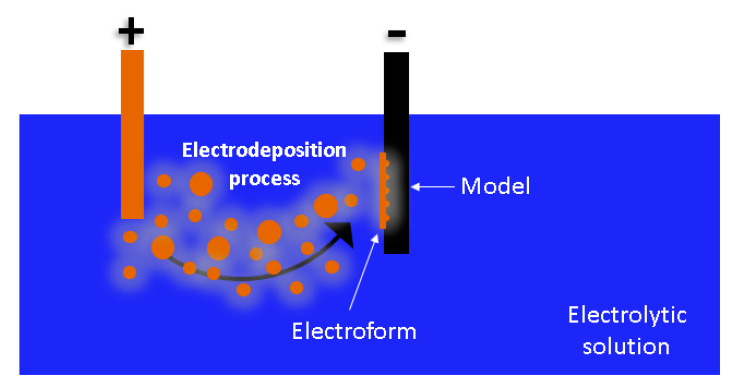
Schematic illustration of electroforming process.

Electroforming is an electrochemical process that is defined as “the production or reproduction of articles by electrodeposition upon a mandrel or mold that it is subsequently separated from the deposit” [27]. Figure 1 schematically shows the process. 

Electroforming requires: an anode (material to be deposited); a cathode (element which receives the deposition and has the geometry to be reproduced); a tank with an electrolytic solution (ion transmission element); and an electric current that is responsible for triggering the electrochemical process. The anode (+) and cathode (−) must be submerged in the solution and must be conductive. Metal deposition generates a shell which forms a piece by itself as a copy of the model. The model can be defined as the negative form to be generated and the electrodeposition shell the positive form. Electroforming is an accumulation process atom by atom theoretically, so it accuracy depends of model design [28]. 

Deposition takes place over time and depends on bath conditions and process parameters. The thickness of pieces increases with the length of the process. It is able to reach values of up to 25 mm in good reproduction conditions [29]. It has the ability to reproduce great dimensional accuracy in three-dimensional geometries, so it has experienced renewed interest given the existing demand for new working procedures [30]. A specialization of the process called micro-electroforming has emerged [31]. It uses special systems such as sources of pulsating energy with polarity inversion [32] or magnetic or ultrasonic electrolytic bath agitation systems [33]. The Integrated and Advanced Manufacturing research Group of the University of Las Palmas de Gran Canaria (ULPGC) has extensive previous experience in the use of special parameters and conditions for electroforming [34,35].

Additive manufacturing is the general term for technologies that create three-dimensional objects by successively adding material based on CAD modelling [36]. Some authors have explored different technologies for the direct electrode manufacturing [37,38]. Tank photopolymerization is one of the oldest techniques. In this process, a liquid photopolymer located in a tank is selectively cured by the action of light [39]. The most common way to work is using an ultraviolet light (UV) system for curing surfaces. The UV light source distinguishes both variants of this process: stereolithography (stereolithography, SLA) and digital light processing (DLP). In DLP technology, a digital micro mirror (DMD) device can be used to project ultraviolet light [40] or a liquid crystal display (LCD), which lights up the geometry to be reproduced [41]. In both modes, the photo reactive materials are polymerized layer by layer. 

Sputtering is the erosion of solid surfaces during the bombardment with energy ions [42,43]. One of its applications, cathodic spraying, is the deposition of thin films on the material surface with ion beam and plasma (PSD and IBSD respectively) [44,45].

Recently Radziejewska et al. have used sputtering (magnetron sputtering) to apply wear-resistance coatings to WC-Co insert of commercial cutting tools. They applied WB_2_ and (W, Ti) B_2_ borides. Their study determined that coatings deposited on WC–Co substrate are smooth and very hard. In one of their tests (turning of difficult-to-cut 304 stainless steel), the W–B coated tool showed better wear resistance than the uncoated tool. Flank wear was smaller by 30% [46].

This work shows the first results of the combined technology AMSME, in application to manufacture copper electrodes for electrical discharge machining processes. Also, is used to texturing aluminium alloys for aeronautical use. An experimental methodology applied to Al-Cu UNS A92024-T3 aluminium alloy test parts, using the SEDM process is proposed. Aluminium alloys have been used in the aeronautical industry, because of their lightness and good relationship between cost and physicochemical properties. Although composites’ use is increasing, it is still used for the manufacture of many parts and components in aeronautical applications. Also, unlimited recycling capacity of aluminium is important to preserve environment. The mechanical properties of these alloys can be improve by applying precipitation hardening treatments [47,48].

Copper electrodes with a functional texture based on a high relief matrix of semi spheres of 2 mm diameter, distributed throughout its surface were used. These shapes cause difficulties in thickness uniformity during electroforming manufacturing. To achieve good results, a laboratory equipment was developed that could be adapted to the combination of DLP additive manufacturing and electroforming.

To advance in the study of surface texturing, it is necessary not to have limitations in design and manufacturing of new geometries. These limitations have been imposed by costs or by the technological limits of both conventional and unconventional machining processes. A new way to work is needed, AMSME method could be an alternative. The goal is to obtain the electrodes and ensure that they are effective for the use in the SEDM process. A positive result raises a new way of approaching functional texturing. The limitations of geometries and shapes for machining electrodes would be left behind thanks to the use of 3D print. High-quality functionals models in detail reproduction could be obtained with low-cost technology, and not just for texturing. It could be a real alternative to manufacturing commercial electrodes for SEDM. In addition, this work could show accessible manufacturing without large factories or machinery. Impossible parts even on a micro scale can be achieved with few errors.

Morkovkin et al. established that the most important factor that limits the growth of industrial production are associated with the lack of resources and the lack of new technologies. The approach of these technologies generates competitive improvement in companies and goods [49].

## 2. Materials and Methods

The work methodology, including texturing, is done in four stages. The last stage is the one in which the work is carried out on the surfaces. To be successfully, the first three must be able to make a tool that works.

### 2.1. Functional Model Manufacturing

Additive manufacturing allows facing the design of geometries until now unreachable. It frees the designer and allows design for manufacturing and assembly (DFMA) to be approached in a different way. However, it is important to master the technology to know its limitations. In this work, this first stage will establish technological limit to geometries to be reproduced.

A DLP/LCD 3D printer machine was used to manufacture the functional model (Wanhao Duplicator 7 (D7), Wanhao 3D printer, Jinhua, China) which emits UV light through an LCD display with a wavelength of 405 µm. This machine projects successive masks in bottom-up mode on the resin found in the tank. It has a pixel resolution of 47 × 47 nm and allows a minimum layer thickness of 30 µm. A polymeric material is used for printing (Monocure 3D Rapid, Monocure Pty Ltd., Sydney, Australia). Table 1 shows the most important parameters of the process.

**Figure 2:**
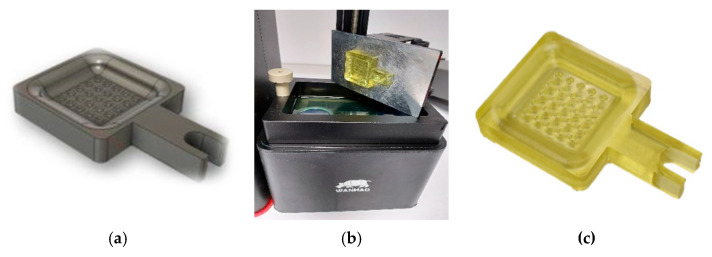
Functional model. (**a**) Modelling (**b**) Manufacturing. (**c**) Result.

Figure 2 shows the functional model used. It is a square geometry element of 38 × 38 mm with a functional surface composed by 25 semi spherical cavities with diameter of 2 mm, with rounding of the radius edge 0.5 mm and pitch 4 mm. 

### 2.2. Sputtering

**Figure 3:**
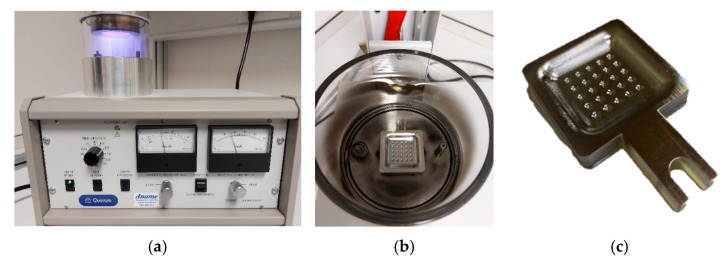
(**a**) Sputtering machine (**b**) Discharge chamber (**c**) Process result.

One of the characteristics which define the electroforming process is the need of active surfaces. These surfaces must be conductive where we want metal deposition. The non-conductive nature of the photosensitive resin of the manufactured model needs to add an intermediate procedure to metallize it. A gold-palladium spraying process (sputtering) with luminescent discharge in the presence of argon is used to do the surface treatment. Figure 3a shows the used equipment (SC7620 Mini Sputter Coater, Quorum Technologies Ltd., Kent, UK), and Figure 3b shows its discharge chamber. Based on the manufacturer’s recommendations, the used parameters were: Process current: 18 mA; Exposed time: 120 s; Voltage: 1 kV. Using these parameters, a layer thickness of 367 Å is achieved. Three depositions were made with a thickness of approximately 1 nanometre to ensure the coating of all functional geometry. Figure 3c shows that the coating reproduces all model surface details. Perpendicular faces need to be masked to avoid particle cloud effect. They could coat these areas and turn them into active surfaces. This creates the possibility of electrodeposition on non-functional surfaces and lower quality deposition.

To verify model results, visual inspections were made with a microscope (MitutoyoTM-1005B, Mitutoyo Corporation, Kanagawa, Japan), and electrical conductivity was checked at different points on the functional surface. It was intended to confirm the entire surface was coated, the deposition has not affected the geometry to be reproduced and the surface was active. The electrical resistance was quantified and values between 160 and 230 Ω were obtained.

**Figure 4:**
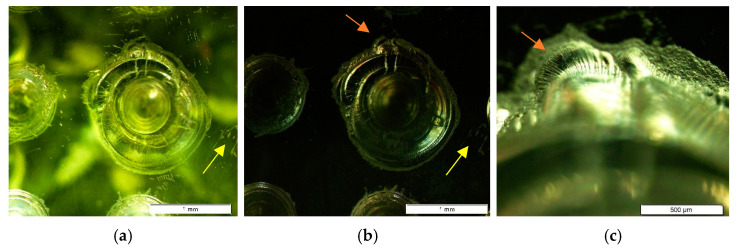
Surface inspection—microscope view (**a**) Before sputtering (20×) (**b**) After sputtering (20×) (**c**) Coating quality (40×).

Figure 4 shows the result of the microscopic view during the inspection process. It can be observed that the sputtering process reproduces both the surface texture and geometric details of the model. The images correspond to a piece with manufacturing defects. It has been used to show how metallizing covers imperfections. Even slight scratches caused by handling (yellow arrow) have been reproduced in detail. Figure 4c shows the coating of the defects in more detail. Sputtering does not modify the geometry, due to the deposited layer works on a smaller scale than the details to be reproduced.

### 2.3. Electroforming

**Figure 5:**
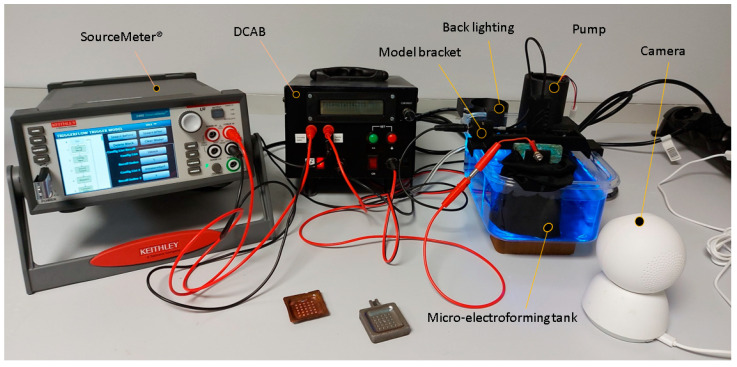
Electroforming equipment.

The metallized resin model was already showed the proposed geometry, then we needed the electroforming to be able to reproduce it. To made it possible an own manufacturing equipment was developed. Figure 5 shows the electroforming equipment in working conditions. It is composed of several elements which manage the different process parameters. Its main components are: Power supply; Model part support system; Electrolytic bath agitation system; Automatic electrolytic bath agitation control device; Monitoring system. These are detailed below.

1. The power supply is the KEITHLEY 2460 SuourceMeter^®^ (Keithley Instruments, Cleveland, OH, USA). This instrument can make real-time measurements of amperage, voltage, and resistance. At the same time, it can provide power with configurable output in voltage or amperage, according to the process needs. It can program electrical cycles and stores readings of the process electrical parameters. These can be used for further analysis.
2. A model holder system is responsible for place and support of the functional model. It has a three-axis movement to allow the adjustment of the distance between anode and cathode, and its orientation.
3. The agitation system consists of a submersible centrifugal pump with a maximum drive flow rate of 2.88 L/min. It has a collector for the distribution of flow with 5 outlet points. This collector is detachable, allows experiments to be conducted under laminar flow or turbulent flow conditions, according to research needs.
4. The automatic electrolytic bath agitation control device (DCAB) is an own design and development device. It manages the electrolytic bath system. It has a manual operation mode and a programmable automatic mode. In automatic mode the system compares the value of current intensity, which circulates through the circuit and the current intensity of setpoint. Based on this comparison, it adjusts the power value of the submerged pump to increase or decrease the recirculation flow of the electrolytic bath.

**Figure 6:**
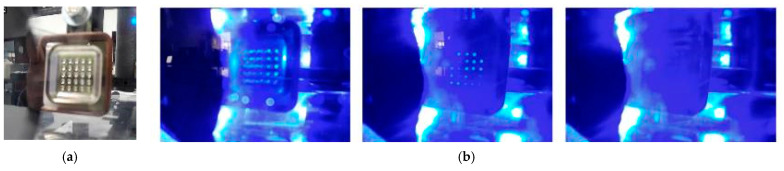
(**a**) Smartphone application’s image (**b**) Electroforming sequence seen from the surveillance system.

The equipment has a monitoring system, which allows to track the electrodeposition in real time. It has continuous recording mode and the possibility to obtain images at any time of the process. It includes a led backlight system that improves the display of the deposition process. The process progress can be tracked at any time and everywhere by a smartphone application. Figure 6a shows the image quality before the dive. Figure 6b shows a images sequence of the electroforming process.

The deposition evolution can be observed until the complete geometry definition. LED backlighting use allows one to identify bright points, which indicates uncompleted cavity backgrounds. As the process progresses you can see which zones are preferred during deposition. This is very interesting to understand the way the part is constructed. Finally, the absence of bright points indicates that the surface has been coated. The recording review allows to set the time interval in which this occurred, providing information relevant to the process optimization.

Test procedure is designed to run in four stages, under controlled laboratory ambient conditions with a temperature of 21 °C and relative humidity below 70%. Work sequences are performed in different times, by setting the current intensity and voltage values according to process needs. These needs were understood after the analysis of electrical data from many experiments. The first stage made a fine deposition which serves as a support and generated uniformity. A low intensity value was set to control growth in more receptive areas. Stages 2 and 3 were performed with a constant voltage to allow progressive details’ formation. In this way, the process spontaneously used the amperage it needed. The last stage was for growth. High amperage was applied to achieve thickness build-up. The operational parameters control of the process is executed by the programmable power supply, and the agitation control of the electrolytic bath by the *DCAB.* A monolithic piece of 100 mm × 50 mm × 10 mm Cu-P alloy is used as an anode. The anode is immersed in an electrolytic bath composed by a copper sulphate acid solution (Bright copper plating bath CU 501, Heimerle-Meule Group, Pforzheim, Germany). The test setup was done under the following conditions: Agitation system turned on under laminar flow conditions, DCAB in automatic mode, 1.1 L solution volume, 1.09 solution PH, Distance between anode and cathode 100 mm and model orientation 0°. Table 2 shows the duty cycle parameters. 

### 2.4. Tooling Design for Adapting the Electrode to a Commercial EDM Electrode Holder

**Figure 7:**
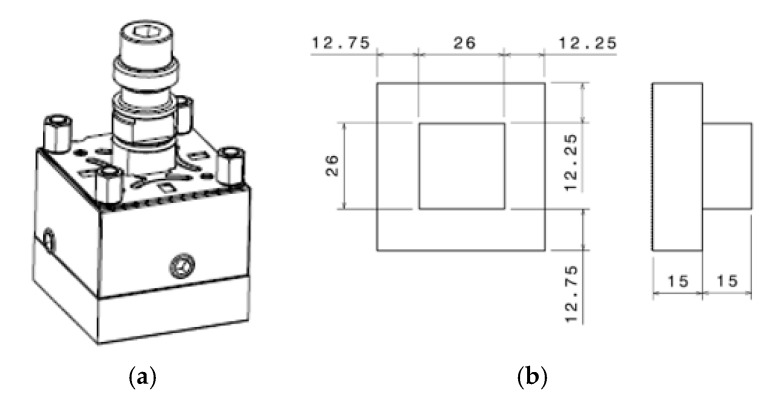
(**a**) EROWA ER-009222 electrode holder. (**b**) Electrode dimensions.

The SEDM test equipment has an electrode holder called EROWA ER-009222 (UCA, Puerto Real, Cádiz, Spain). It was designed to house a graphite electrode as a wear tool (Figure 7a).

**Figure 8:**
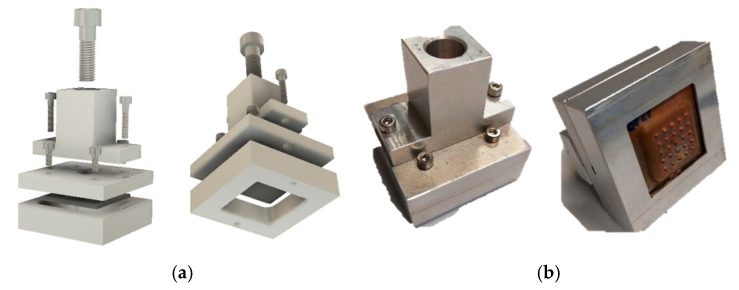
(**a**) Shell holder modelling. (**b**) Shell holder manufactured.

**Figure 9:**
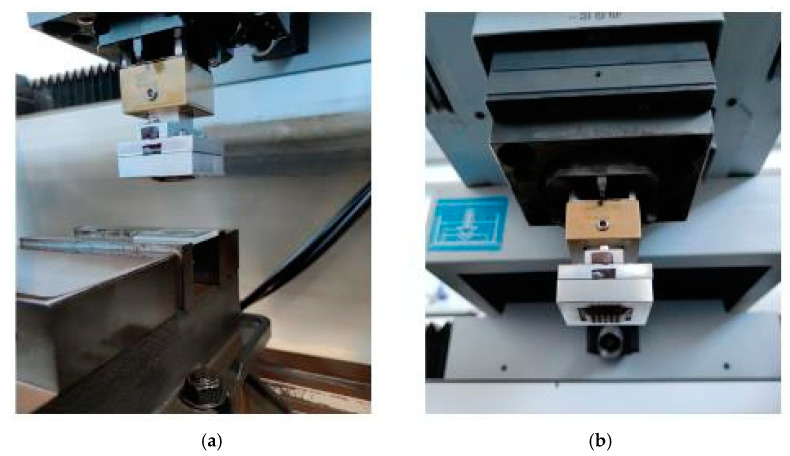
(**a**) Electrode and test piece. (**b**) Shell, Shell holder, and Electrode holder.

A shell holder was developed to perform the tests with the electroformed copper shell. It can be attached to the EROWA ER-009222, with the shape and dimensions shown in Figure 7b. A detachable element consisting of 4 pieces was designed. These pieces are joined together by bolted mechanical fixation, as shown in Figure 8a. All elements of the assembly were manufactured in EN-AW5083 H111 aluminium to ensure electrical conductivity. Figure 9 shows the electrode mounted on the machine under service conditions.

SEDM texturing tests were performed on an Al-Cu UNS A92024-T3 aluminium alloy sheet. The A92xxx series aluminium alloys account with a high copper content. Copper improves aging hardening behavior. The alloys also have good mechanical strength. The UNS A92024-T3 alloy has specific applications in the aeronautical industry. It is used in areas under fatigue efforts, such as the fuselage of pressurized cabs and the coating lining of the lower part of the wing, among others [50]. An example of its application is found on the fuselage panels of the mythical Lockheed C-130 Hercules transport aircraft [51].

The sample texturing process was performed at the same erosion depth, which was set at 0.25 mm. Three tests were made with three different surface qualities. Determine the electrode abilities for different finishing is needed. Erosion technology was selected with minimum wear criteria to increases the copper electrode’s life. The used parameters are detailed in Table 3.

### 2.5. Validation Method

We review three aspects to establish the success level of the procedure: electroformed part mass; geometric and surface quality; Shell thickness at edges, functional area, and backgrounds of semi-spherical cavities. 

The mass measurement of the electroformed part is performed with an analytical balance (Mettler-Toledo AB204-S, Mettler-Toledo LLC, Columbus, OH, USA). 

**Figure 10:**
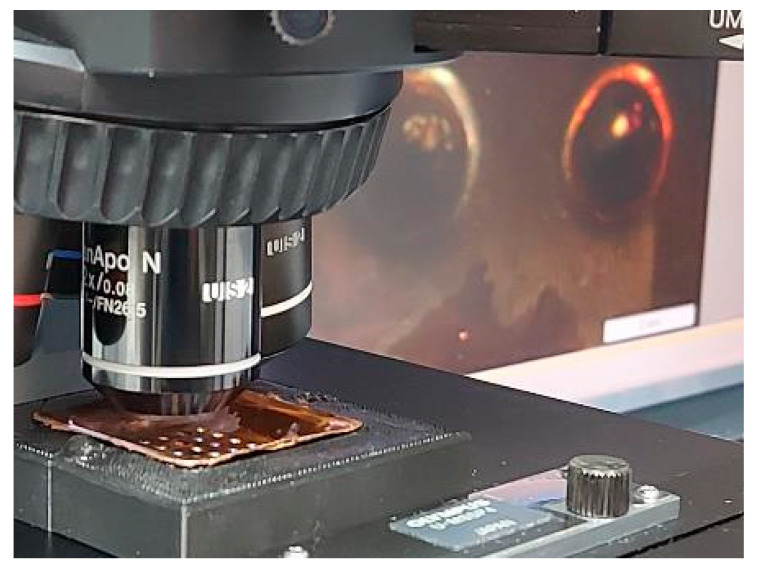
Microscope inspection process.

A microscopic inspection of the copper shell is performed to evaluate the replicate detail’s ability. We used a measuring electron microscope (Olympus BX51, Olympus Corporation, Tokyo, Japan) with a magnification of 20× (Figure 10).

**Figure 11:**
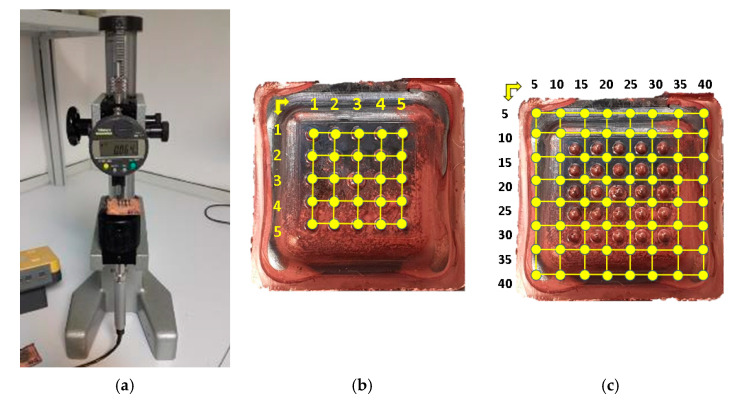
(**a**) Measurement process. (**b**) Cavities measurement points. (**c**) Coordinates of surface measurement points.

A measurement process has been performed at different points to check the thicknesses of the parts. The measurements were made with a branded mechanical comparator (Mitutoyo ID-C112B, Mitutoyo Corporation, Kanagawa Prefecture, Japan) mounted on a comparator verification bench. This equipment was calibrated and has a calibration certificate (No862), issued by the Metrology and Calibration Service (SMC) of the University of Las Palmas de Gran Canaria. Figure 11a shows the set under service conditions. Figure 11b,c show the positions of these measurement points.

## 3. Results and Discussion

### 3.1. Electroforming Results

The mass of the electroformed shell allows us to establish an economic reference with respect to electrodes manufactured by conventional methods. These, usually use monolithic pieces of material from which geometry is then extracted, therefore they need more material. The measurement result was 11.9775 gr. This value was lower than any other electrode manufactured by conventional methods.

**Figure 12:**
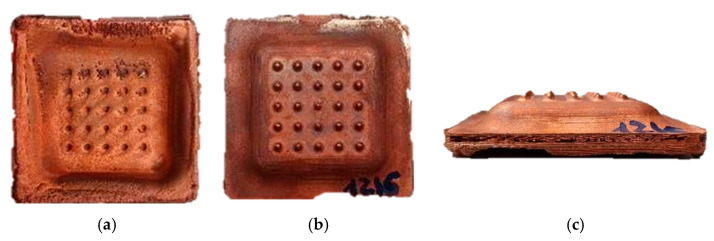
Finished shell (**a**) Non-functional face. (**b**) Functional face. (**c**) Thickness view.

Figure 12 shows several views of the finished part. The geometry has been completed and presented an excellent finish on the functional face. The finish on the non-functional surface is not relevant, because it will be filled with a 10 mm thick layer of epoxy resin. This will give it enough volume and rigidity to fit into the tool holder and to withstand the stresses of the SEDM process.

**Figure 13:**
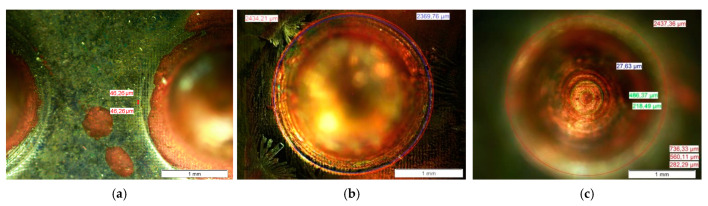
Microscope view (20×) (**a**) Shell Surface. (**b**) Cavity surface. (**c**) Cavity bottom.

The inspection with the measuring electron microscope confirms good results. Figure 13 shows the surface part appearance in different areas. The whole geometry has been completed successfully, including the bottom and the surface of the semi-spherical cavities. Several details on a microscopic scale were reproduced, such as the pixels of the LCD screen (Figure 13a) or the steps of the resin layers (Figure 13c), whose values were already indicated in sections before. Although the geometry exposed in this work was used for the set-up and evaluation of the abilities of the equipment, the results showed the potential for manufacturing microdetails.

Table 4 shows the average values resulting from measuring thicknesses on the parts’ surfaces. Table 5 shows the average values resulting from measuring thicknesses at the bottoms of semi-spherical cavities. The average value for the full functional area is 991.6 µm and the minimum value is 383 µm. The average value for the bottoms of the semi-spherical cavities is 406 µm and the minimum value is 328 µm. The average deposition in the backgrounds has lower values, due to the difficulty of deposition in the incoming geometries. In shaded areas, the current decreases, due to an increase in resistance.

**Figure 14:**
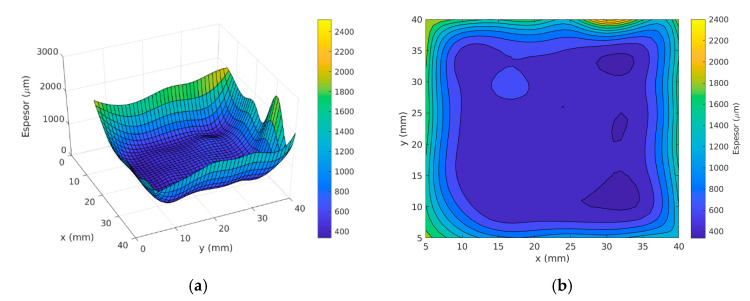
(**a**) 3D thickness Map. (**b**) Surface thickness distribution.

Figure 14 shows the results graphically. It is observed that the thickness increases at the borders, due to current concentration. Also produces a smaller thickness at the bottom of the spherical cavities.

### 3.2. SEDM Results

We did a visual inspection and weighing of shells and electro-eroded test parts to verify the ability of the electroformed micro tool to do machining operations under working conditions.

From a geometric point of view, the electrode wear and the quality of the eroded surface was inspected. To do this, a stereoscopic microscope (Nikon SMZ800, Nikon Corporation, Tokyo, Japan) equipped with CCD camera with a 20× magnification was used.

**Figure 15:**
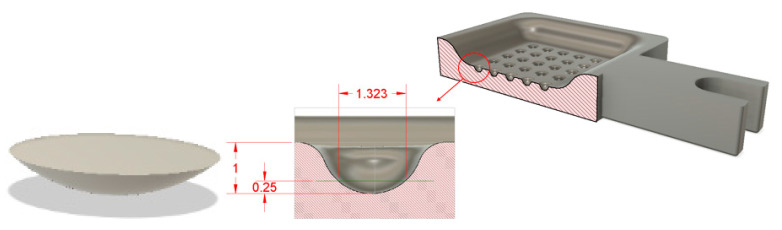
Theorical erosion semi sphere.

**Figure 16:**
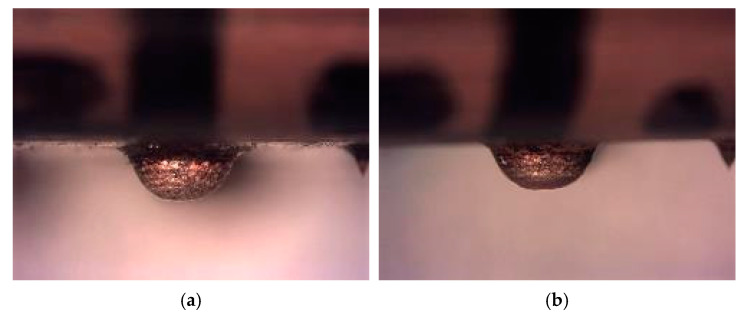
Semi sphere 1.1. (**a**) Before erosion process (**b**) After first erosion process.

We studied the three semi-spheres of the shell located in the first row (1.1, 1.2 and 1.3), and their corresponding eroded prints. Figure 15 shows theoretical erosion geometry and its geometric parameters. Figure 16b shows erosion effects for VDI 0 test. Good shell ending is observed. Semi-spherical geometry keeps its shape after this first process. 

**Figure 17:**
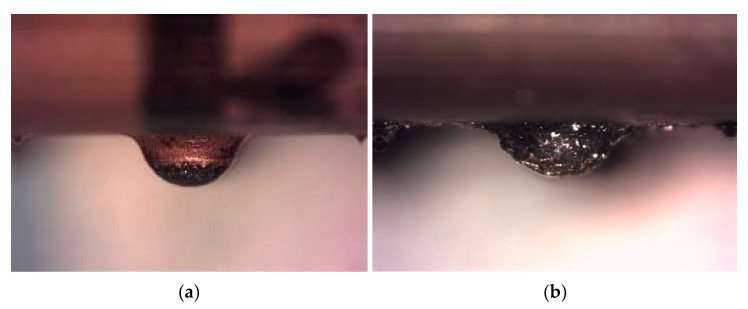
Semi sphere 1.1 Result after erosion. (**a**) Test 2 (**b**) Test 3.

Figure 17a shows second test erosion effects with VDI 27. Good shell outcome is also observed. Semi-spherical geometry loses some uniformity, but still preserves integrity after this second process. The eroded area reaches approximately one third of semi sphere height, which coincides with the depth value used as a setpoint (0.25 mm)

Figure 17b shows third test erosion effects with VDI 40. Significant degradation under roughing conditions is observed. Semi-spherical geometry loses its integrity after this third process and the eroded area reaches the surface of the part. 

**Figure 18:**
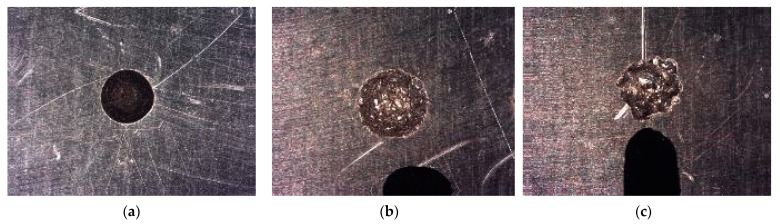
SEDM results. (**a**) VDI 0 (**b**) VDI 27 (**c**) VDI 40.

Figure 18 shows the effects of electro erosion on the aluminium parts from the three tests. The shell’s geometry shows a good definition, and a good cavity edges finish. Less quality shell’s geometry is produced when SEDM process is more demanding.

At the end of the analysis, we weigh test pieces and shells to verify the wear level. This process was made with a precision balance (OHAUS Pioneer, OHAUS Europe GmbH, Nänikon, Switzerland) with a resolution of 0.0001 g. Table 6 shows the result of the weighing process average values. Data collection was performed before and after each test. Mass decrease is a reference to show the wear level. The aluminium particles adhesion to electrode and dielectric fluid absorption on the copper shell suggest complementary mass increases, which are very difficult to evaluate, and therefore to compensate.

After the first test, the electroformed shell exhibits a mass increase. Due to the porosity of copper, the absorption of dielectric liquid after the electro erosion process is higher than material loses from wear. So, the success of the process cannot be estimated from shell mass measure. However, the results of visual inspection show a very little wear. Therefore, the electrode shows good behavior to work with low VDIs.

After the second and third test, there is no relevant variation in mass, which does not correspond to what is observed in the visual inspection. The mass increase produced by the adhesion of aluminium particles which comes from the machined surface during the SEDM process, explained this difference. Also, there is a dielectric fluid absorption from a greater porosity of the electrode surface. This phenomenon increases as it receives more discharges. 

The Al-Cu alloy part surface has suffered a mass decrease of 0.0141 g in the first test, and a decrease of 0.0284 g in the second. Its shows a logical evolution, due to more severe conditions of the second SEDM process. However, this evolution is reversed in test 3. The excessive tool wear results in reduced machinability. We selected the mass as reference parameter of the test parts to make a comparison between the theoretical and the real wear, because it is easy to measure. This method requires the calculation of the theoretical volume of the wear geometry. As shown in Figure 15, we used a CAD tool to do this. Considering the erosion depth at 0.25 mm, the theoretical volume of the wear geometry results in 0.18 mm^3^.

**Figure 19:**
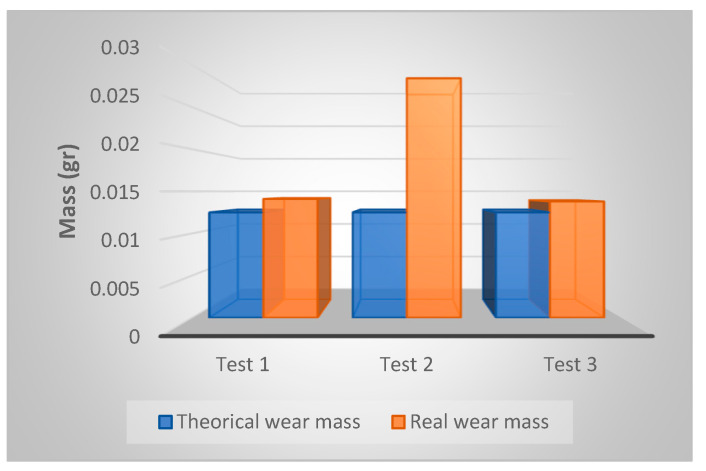
Graphical representation of wear mass comparative.

Theoretical wear masses were calculated from the 25 semi spherical erosion geometries, which form the functional surface. Real wear masses were obtained from measurement processes. Table 7 shows the comparison. Figure 19 graphically represents this comparison.

**Figure 20:**
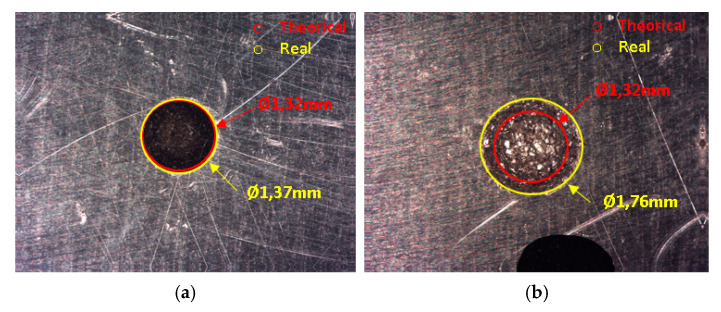
Geometrical comparison. (**a**) VDI 0 (**b**) VDI 27.

As a result of test 1, wear values are very similar to those theoretically expected. As shown in Figure 20a a good reproduction of geometry supports this result. The electro-erode trace diameter (Ø1.32 mm) is very similar to the theorical diameter (Ø1.37 mm). The difference is about 50 µm. Test 2 shows important differences. The wear mass is more than twice as high as the theoretical mass. The erosion exceeds the limits of the predicted geometry under these conditions. The shell maintains its integrity but loses dimensional accuracy in its ability to reproduce. Figure 20b shows the comparison between the predicted diameter and the achieved diameter. The difference is about 440 µm. In test 3 the comparison between theoretical wear mass and real wear mass is approaching again. However, this result does not add value, because the rapid degradation of the electrode does not allow to obtain high quality erosion. Therefore, the match of the mass values is anecdotal. 

## 4. Conclusions

We did the texturing of Al-Cu UNS A92024-T3 aeronautical aluminium alloy using a penetration electro erosion process (SEDM) with copper electrode. A combined process (AMSME) was used for electrode manufacturing. Furthermore, it was necessary to develop a laboratory equipment to manufacture this electroformed part with microscopic scale details. The following conclusions can be drawn from the conducted research:

- The AMSME process has demonstrated great potential in shaping functional surfaces for its/the use as SEDM electrodes.
- The SLA/DLP process obtains high-quality functional models in the reproduction of details with low-cost technology. In this way, its cost, one of the main limitations of the manufacture of these electrodes, can be overcome.
- The sputtering process ensures the correct coating of the entire surface and generates conductivity in the active zones.
- The developed equipment for micro-electroforming operations shows to be effective in generating copper shells with high level details. The use of different power stages has been decisive in its manufacture.
- Micro-electroformed shell shows good reproduction abilities in its application to SEDM. For high VDIs the finish of the test surfaces is not acceptable, and the electrode deteriorates rapidly. However, its performance for finishing processes with medium-low VDI, has been very good based on criteria of minimum wear. Its shows great potential to do texturing works at an industrial level.
- The surface texturing with electroformed copper electrode could be a great alternative to electrodes of other materials for microscopic-scale work. The machining with the latter is difficult or impossible at this scale.

## Figures and Tables

**Table 1:** Process parameters.

Resin	Exposure Time	Cure Time	Resin Usage	Layers	Print Time
Monocure 3d Clear	10.5 s	4.0 s	10.13 mL	267	1 h 17′23″

**Table 2:** Process stages.

Stage	Current	Voltage	Time
1	100 mA	variable	600 s
2	variable	0.5 V	3600 s
3	variable	1.5 V	3600 s
4	900 mA	variable	64,800 s

**Table 3:** SEDM settings.

VDI	Ra	Current	Voltage	Pulse T.	Pause T.	Reverse T.	Work T.	Erosion T.
(Norma VDI 3402)	(µm)	(A)	(V)	(µs)	(µs)	(s)	(s)	(s)
0	0.10	1	−200	3.2	6.4	0.3	0.3	10,800
27	2.2	5	200	13	6.4	0.3	0.4	2700
40	10	10	80	400	50	0.3	1.5	15

**Table 4:** Surface thickness.

Position (mm)	5	10	15	20	25	30	35	40
5	1884 µm	1397 µm	1058 µm	1047 µm	1195 µm	1121 µm	1312 µm	1223 µm
10	1607 µm	737 µm	432 µm	430 µm	414 µm	383 µm	452 µm	1396 µm
15	1534 µm	534 µm	481 µm	461 µm	521 µm	414 µm	512 µm	1566 µm
20	1654 µm	542 µm	496 µm	517 µm	495 µm	411 µm	557 µm	1730 µm
25	1776 µm	583 µm	528 µm	567 µm	589 µm	440 µm	514 µm	1682 µm
30	1758 µm	558 µm	627 µm	584 µm	539 µm	455 µm	555 µm	1718 µm
35	1881 µm	557 µm	594 µm	650 µm	578 µm	465 µm	527 µm	1586 µm
40	1964 µm	1738 µm	1616 µm	1712 µm	1430 µm	2522 µm	1642 µm	2013 µm

**Table 5:** Cavity bottom thickness.

Position	1	2	3	4	5
1	365 µm	363 µm	488 µm	391 µm	445 µm
2	428 µm	374 µm	328 µm	388 µm	369 µm
3	392 µm	414 µm	364 µm	481 µm	488 µm
4	373 µm	437 µm	463 µm	335 µm	535 µm
5	384 µm	378 µm	427 µm	396 µm	358 µm

**Table 6:** Difference between test parts and shell masses. Average values.

Mass Comparison	Test 1		Test 2		Test 3	
	Electrode	Test Part	Electrode	Test Part	Electrode	Test Part
Before	30.9338 g	33.4904 g	30.9556 g	33.2745 g	30.9557 g	33.4302 g
After	30.9556 g	33.4763 g	30.9557 g	33.2461 g	30.9558 g	33.4164 g
Difference	0.0218 g	−0.0141 g	0.0001 g	−0.0284 g	0.0001 g	−0.0138 g

**Table 7:** Wear mass comparative.

Mass Comparison	Test 1	Test 2	Test 3
Theorical wear mass	0.0125 g	0.0125 g	0.0125 g
Real wear mass	0.0141 g	0.0284 g	0.0138 g
Difference	−0.0016	−0.0159	−0.0013

## Data Availability

The data underlying this article will be shared on reasonable request from the corresponding author.

## References

[1] Sorgato M., Masato D., Lucchetta G. (2017). Effects of machined cavity texture on ejection force in micro injection molding. Precis. Eng..

[2] Ibatan T., Uddin M., Chowdhury M.A.K. (2015). Recent development on surface texturing in enhancing tribological performance of bearing sliders. Surf. Coat. Technol..

[3] Hamilton D.B., Walowit J.A., Allen C.M. (1966). A Theory of Lubrication by Microirregularities. J. Basic Eng..

[4] Evans C.J., Bryan J.B. (1999). “Structured”, “Textured” or “Engineered” Surfaces. CIRP Ann..

[5] Stout K.J., Blunt L. (2001). A contribution to the debate on surface classifications-Random, systematic, unstructured, structured and engineered. Int. J. Mach. Tools Manuf..

[6] Li X.-M., Reinhoudt D., Crego-Calama M. (2007). What do we need for a superhydrophobic surface? A review on the recent progress in the preparation of superhydrophobic surfaces. Chem. Soc. Rev..

[7] Said S.A.M., Al-Aqeeli N., Walwil H.M. (2015). The potential of using textured and anti-reflective coated glasses in minimizing dust fouling. Sol. Energy.

[8] Moronuki N. (2016). Functional Texture Design and Texturing Processes. Int. J. Autom. Technol..

[9] Çakīr O., Yardimeden A., Ozben T., Kilickap E. (2007). Chemical machining. Arch. Mater. Sci. Eng..

[10] Harris W.T. (1976). Chemical Milling. The Technology of Cutting Materials by Etching.

[11] Ao S., Qin X., Li K., Luo Z. (2021). Effects of process parameters on jet electrochemical machining of SiC particle-reinforced aluminum matrix composites. Int. J. Adv. Manuf. Technol..

[12] Joshi G.S., Putignano C., Gaudiuso C., Stark T., Kiedrowski T., Ancon A., Carbone G. (2018). Effects of the micro surface texturing in lubricated non-conformal point contacts. Tribol. Int..

[13] Simão J., Aspinwall D.K., Wise M.L.H., El-Menshawy M.F. (1994). Mill roll texturing using EDT. J. Mater. Process. Technol..

[14] Prasad K.N., Syed I., Subbu S.K. (2019). Laser dimple texturing–applications, process, challenges, and recent developments: A review. Aust. J. Mech. Eng..

[15] Ding Q., Wang L., Hu L. (2015). Tribology Optimization by Laser Surface Texturing: From Bulk Materials to Surface Coatings. Laser Surface Engineering: Processes and Applications.

[16] Li Z., Bai J., Tang J. (2018). Micro-EDM method to fabricate three-dimensional surface textures used as SERS-active substrate. Appl. Surf. Sci..

[17] Chung D.K., Shin H.S., Park M.S., Kim B.H., Chu C.N. (2011). Recent researches in micro electrical machining. Int. J. Precis. Eng. Manuf..

[18] Lazarenko B.R., Lazarenko N.I., Brutcher H.E. (1950). Electric Spark Method for the Machining of Metals.

[19] Kumar S., Singh R., Singh T.P., Sethi B.L. (2009). Surface modification by electrical discharge machining: A review. J. Mater. Process. Technol..

[20] DKim M., Lee I., Kim S.K., Kim B.H., Park H.W. (2016). Influence of a micropatterned insert on characteristics of the tool–workpiece interface in a hard turning process. J. Mater. Process. Technol..

[21] Koshy P., Tovey J. (2011). Performance of electrical discharge textured cutting tools. CIRP Ann..

[22] Xu X., Guo P., Tiong LC O., Zuo X., Li X., Lee K.R., Wang A. (2020). Role of dimple textured surface on tribological properties of Ti/Al-codoped diamond-like carbon films. Thin Solid Films.

[23] Graham E., Park C.I., Park S.S. (2013). Fabrication of micro-dimpled surfaces through micro ball end milling. Int. J. Precis. Eng. Manuf..

[24] Pal V.K., Choudhury S.K. (2016). Fabrication of texturing tool to produce array of square holes for EDM by abrasive water jet machining. Int. J. Adv. Manuf. Technol..

[25] Lu P., Wood R.J.K., Gee M.G., Wang L., Pfleging W. (2018). A Novel Surface Texture Shape for Directional Friction Control. Tribol. Lett..

[26] Liew K.W., Kok C.K., Efzan M.N.E. (2016). Effect of EDM dimple geometry on friction reduction under boundary and mixed lubrication. Tribol. Int..

[27] Parkinson R. (1998). Electroforming—A Unique Metal Fabrication Process.

[28] Hernández P. (2015). Electroforming Applied to Manufacturing of Microcomponents. Procedia Eng..

[29] Wook K., Yul K., Sang J. (2010). Method of Duplicating Nano Pattern Texture on Object’s Surface by Nano Imprinting and Electroforming. U.S. Patent.

[30] McGeough J.A., Leu M.C., Rajurkar K.P., de Silva A.K.M., Liu Q. (2001). Electroforming Process and Application to Micro/Macro Manufacturing. CIRP Ann..

[31] Ding J., Zhai Z., Hejijun C., Jiawen L., Wang X., Huang W. Micro Device Mould Fabrication Based on Two-Photon Polymerization and Electroforming. Proceedings of the 2010 IEEE 5th International Conference on Nano/Micro Engineered and Molecular Systems.

[32] Wong K.P., Chan K.C., Yue T.M. (1999). A study of surface finishing in pulse current electroforming of nickel by utilizing different shaped waveforms. Surf. Coat. Technol..

[33] Weinmann M., Jung A., Natter H. (2013). Magnetic field-assisted electroforming of complex geometries. J. Solid State Electrochem..

[34] Hernández P., Benítez-Vega A.N., Díaz-Padilla N., Ortega-García F., Socorro-Perdomo P., Marrero-Alemán M.D. (2017). Design and manufacture of structured surfaces by electroforming. Procedia Manuf..

[35] Benítez A., Ortega F., Monzón M., Hernández P., Marrero M., Carrión C. Desarrollo de Electrodos EDM a Partir de Fabricación Aditiva de. Electroconformado. Proceedings of the XVIII Congreso Nacional de Ingeniería Mecánica.

[36] American Society for Testing (2015). Standard Terminology for Additive Manufacturing—General Principles—Terminology.

[37] Alan A., Michael D.P., Charles C.R. (1996). Using rapid prototyping to produce electrical discharge machining electrodes. Rapid Prototyp. J..

[38] Gillot F., Mognol P., Furet B. (2005). Dimensional accuracy studies of copper shells used for electro-discharge machining electrodes made with rapid prototyping and the electroforming process. J. Mater. Process. Technol..

[39] Gibson I., Rosen D.W., Stucker B. (2010). Additive Manufacturing Technologies.

[40] Kadry H., Wadnap S., Xu C., Ahsan F. (2019). Digital light processing (DLP) 3D-printing technology and photoreactive polymers in fabrication of modified-release tablets. Eur. J. Pharm. Sci..

[41] Wu X., Xu C., Zhang Z. (2021). Flexible film separation analysis of LCD based mask stereolithography. J. Mater. Process. Technol..

[42] Smentkowski V.S. (2000). Trends in sputtering. Prog. Surf. Sci..

[43] Handoo A.K., Ray P.K. (1992). Sputtering yield of chromium by argon and xenon ions with energies from 50 to 500 eV. Appl. Phys. A.

[44] Auciello O., Kingon A.I., Krupanidhi S.B. (1996). Sputter Synthesis of Ferroelectric Films and Heterostructures. MRS Bull..

[45] Cuomo J.J., Rossnagel S.M., Kaufman H.R. (1989). Handbook of Ion Beam Processing Technology.

[46] Radziejewska J., Psiuk R., Mościcki T. (2020). Characterization and Wear Response of Magnetron Sputtered W–B and W–Ti–B Coatings on WC–Co Tools. Coatings.

[47] Grilli R., Baker M.A., Castle J.E., Dunn B., Watts J.F. (2010). Localized corrosion of a 2219 aluminium alloy exposed to a 3.5% NaCl solution. Corros. Sci..

[48] Badawy W.A., Al-Kharafi F.M., El-Azab A.S. (1999). Electrochemical behaviour and corrosion inhibition of Al, Al-6061 and Al–Cu in neutral aqueous solutions. Corros. Sci..

[49] Morkovkin D., Gibadullin A., Safarov B., Alpatova E. (2020). Definition of factors limiting the growth of industrial production. IOP Conf. Ser. Mater. Sci. Eng..

[50] Vázquez J.E.D., Bárcena R.B., Ares P.F.M., Miras J.M.G., Marcos M., Pedemonte F.J.B. Influencia del Tiempo de Exposición al Medio Corrosivo en la Resistencia a la Tracción de Aleaciones de Aluminio.

[51] Merati A. (2005). A study of nucleation and fatigue behavior of an aerospace aluminum alloy 2024-T3. Int. J. Fatigue.

